# A Clinical Prediction Rule for Predicting the Health-related Quality of Life after 5 Months in Patients with Knee Osteoarthritis Undergoing Conservative Treatment

**DOI:** 10.1298/ptr.E10296

**Published:** 2024-11-28

**Authors:** Shunsuke YAMASHINA, Tetsuya AMANO, Shigeharu TANAKA, Yu INOUE, Ryo TANAKA

**Affiliations:** 1 Department of Rehabilitation, Taira Hospital, Japan; 2 Department of Environmental Medicine and Public Health, Faculty of Medicine, Shimane University, Japan; 3 Graduate School of Humanities and Social Sciences, Hiroshima University, Japan; 4 Department of Physical Therapy, Faculty of Health and Medical Sciences, Tokoha University, Japan; 5 School of Rehabilitation, Kanagawa University of Human Services, Japan; 6 Department of Human Science, School of Human Science, Kibi International University, Japan

**Keywords:** Clinical prediction rule, Knee osteoarthritis, Machine learning, Health-related quality of life

## Abstract

Objective: This study aimed to derive a clinical prediction rule (CPR) that can predict changes in health-related quality of life at 5 months for patients with knee osteoarthritis (KOA) undergoing conservative treatment. Methods: Patients with KOA receiving physical therapy and exercise therapy at an outpatient clinic were included in this study. The basic characteristics, medical information, and motor function test results were recorded at baseline. The primary outcome measure was the change in the Japan Knee Osteoarthritis Measure (JKOM) 5 months after the baseline measurement. A decision tree analysis was performed with the basic characteristics, medical information, and the motor function test results as the independent variables and the changes in the JKOM after 5 months (≥8 in the improved groups) as the dependent variable. Results: Analyzed data from 87 patients. The variables included the visual analog scale score, bilateral KOA, 5-m walk test, JKOM, and body mass index. Six CPRs were obtained from the terminal nodes. Accuracy validation of the model for the entire decision tree revealed an area under the receiver operating characteristic curve of 0.87 (validation data: 0.83), a positive likelihood ratio of 2.6, and a negative likelihood ratio of 0.1. Conclusion: This CPR is an inspection characteristic that can exclude the possibility of the occurrence of an event based on a negative result. However, since the results of this study represent the first process of utilizing the CPR in actual clinical practice, its application should be kept in mind.

## Introduction

The prevalence of knee osteoarthritis (KOA) is increasing in aging populations, and the number of patients with KOA in developed countries is predicted to double by 2031^1)^. The healthcare expenditure of patients with osteoarthritis is estimated to be four times higher than that of those without osteoarthritis. Furthermore, healthcare expenditure tends to increase as the patient ages^2)^. Therefore, pressure on medical costs is inevitable unless we develop highly effective treatment interventions and optimize treatment choices through evaluation. The first choice of treatment for patients with KOA is follow-up with conservative treatment, such as pharmacotherapy, exercise therapy, and patient education^3^^,^^4)^. Several guidelines recommend the implementation of exercise therapy and patient education as it is associated with a low risk of side effects and intervention costs^5–8)^. Identifying cases that could benefit from exercise therapy in advance may facilitate the attainment of effective therapeutic outcomes while lowering the effect of side effects.

Health-related quality of life (HRQOL) assessments have been used to determine the effectiveness of KOA treatment in clinical practice. Among these, the Japan Knee Osteoarthritis Measure (JKOM) is an HRQOL scale that reflects the social and cultural background of Japan^9)^. HRQOL is an important indicator that is used to determine whether total knee arthroplasty (TKA) should be performed in patients with KOA. Notably, compared with that of patients who have not undergone TKA, a decline in HRQOL and daily living functions is observed postoperatively in patients who have undergone TKA^10)^. A lower preoperative HRQOL leads to poorer HRQOL at 6 months postoperatively^11)^. HRQOL assessment plays a role in the decision to perform surgical treatment in patients with KOA. Moreover, it has a negative effect on the postoperative period. Thus, improving HRQOL should be a goal of conservative treatment.

Previous studies have shown that exercise therapy and pharmacotherapy for patients with KOA improve HRQOL to some extent. A report using JKOM, the Western Ontario and McMaster Universities Arthritis Index (WOMAC), Short-Form 36-Item Health Survey (SF-36), and visual analog scale (VAS) as outcomes, reported that 8-week exercise therapy resulted in an improvement of 8–14 points, 5.21–15.62 points, 4.36–7.91 points, and 14.61–23.04 mm in outcome scores, respectively^12)^. However, some patients have shown poor response to exercise therapy^13)^. Lack of clarity regarding which patients can and cannot benefit from exercise therapy can lead to inappropriate treatment choices, which may result in unnecessary medical costs. Therefore, it is important to clarify the criteria for exercise therapy application.

The clinical prediction rule (CPR) is a useful tool for predicting treatment responses. Previous studies have investigated the use of CPR to rule out knee fractures and support the diagnosis of KOA^14^^,^^15)^. In other studies, patients with KOA who were ≤69 years of age, had knee flexion muscle strength of ≤0.36 Nm/kg, and had a VAS score of ≥33 mm were less likely to benefit from usual exercise therapy and could be predicted to be less physically activity^16)^. These studies have indicated that multiple pieces of information can be combined to predict specific events. The use of CPR in patients with KOA is beneficial in that it facilitates the screening of patients likely to benefit from exercise therapy, thereby establishing the criteria for the application of exercise therapy. On the other hand, CPR has not been used to predict HRQOL in patients with KOA receiving conservative treatment. Thus, this study aimed to derive a CPR that predicts the HRQOL at 5 months for patients with KOA undergoing conservative treatment.

## Methods

### Ethical statement

This study was approved by the Research Ethics Committee of Tokoha University and adhered to the tenets of the Declaration of Helsinki (approval number: 2022-501H). Written informed consent and assent were obtained from all participants prior to enrollment.

### Study design

The study was conducted as a multicenter, eight-center study. The study design was a prospective cohort study. The observation period was set at 5 months. This was based on the fact that the Japanese reimbursement system for musculoskeletal rehabilitation usually sets the period of coverage at 5 months. However, rehabilitation may be performed beyond 5 months in some cases.

### Participants

Patients with KOA receiving physical therapy and exercise therapy at an outpatient clinic who met the following criteria were included in this study: age, 50–90 years; Kellgren-Lawrence classification (K-L classification) categories 1–4; and patients capable of walking ≥10 m by themselves. Patients receiving special therapies or treatment with special equipment (determined based on the details of exercise therapy and physical therapy to avoid major differences in treatment content), patients with neurological disorders (affecting lower-limb muscle strength and walking), and patients with cognitive impairment (affecting the results of the self-administered questionnaires) were excluded.

### Variables

With reference to variables from previous studies^16)^, the following characteristics were recorded at baseline: sex, age, body mass index (BMI), and exercise habits (exercising at least twice a week for at least 30 minutes at a time for at least 1 year, which entailed exercises such as walking and strength training). Data regarding the following medical information were also recorded: the severity of KOA (as assessed using the K-L classification), the severity of pain (as assessed using the visual VAS score, maximum pain at rest and during activity in the past week), bilateral KOA, pharmacological treatment, duration of physical therapy intervention (within 1 month, 1–5 months, 6–11 months, and ≥12 months), physical therapy intervention time (minutes per week), physical therapy start date (0: The period from the date of diagnosis to that of starting physical therapy was less than 1 month, 1: The period from the date of diagnosis to that of starting physical therapy was at least 1 month) and the Self-Efficacy for Rehabilitation Outcome Scale (SER) score. The results of the following motor function tests were also recorded: knee joint extension muscle strength on the fault side, knee joint flexion muscle strength on the fault side, knee joint extension range of motion on the fault side, knee joint flexion range of motion on the fault side, five-times stand and sit test (FTSST), 5-m fastest walk time (as assessed using the 5-m walk test; 5mWT), and HRQOL (as assessed using JKOM). The K-L classification category was higher for patients with bilateral KOA, and the side with more pain was defined as the fault side.

The K-L classification was determined by an orthopedic surgeon using radiographic images and rated on a five-point scale based on the presence of osteophytes and deformities. The scores ranged from 0 to 4, and category 4 was considered the most severe^17)^. SER uses a dedicated patient-oriented questionnaire comprising 12 items scored on a scale of 0 to 10, with a score of 0 for “not at all confident” and 10 for “very confident.” The total score ranged from 0 to 120^18)^. The knee joint muscle strength on the fault side was measured using a handheld dynamometer (μTas F-1; Anima, Tokyo, Japan), with the sensor fixed using a band. The patients were seated with the knee joint at 90° flexion. The maximum isometric contraction of the knee joint extension and flexion muscles was measured in this position. The distance from the center of the knee joint to the sensor was defined as the lever arm length. The values were normalized to the lever arm length and body weight (Nm/kg). The measurements were acquired twice, and the better of the two results was selected as the representative value. The test–retest intraclass correlation coefficient (ICC) for this measurement method ranged from 0.85 to 0.92^19)^. In addition, the inter-rater ICC was 0.93^20)^. The knee joint range of motion on the fault side was measured to determine the maximum range of motion of the knee joint in other motions. The values were recorded using a goniometer in 5° increments. The measurements were acquired twice, and the better of the two results was selected as the representative value. The test–retest ICC for this measurement method ranged from 0.81 to 0.96 ^21)^. Additionally, the inter-rater ICC was 0.72–0.80^22)^. FTSST was performed by instructing the patients to stand up from a chair and sit down as rapidly as they could. This process was repeated five times, and the time required to complete five repetitions was measured. The upper limbs were crossed in front of the chest. The patients were seated both at the beginning and at the end of the test. The chair had a backrest and was 42 cm high. The measurements were acquired twice, and the better of the two results was selected as the representative value. Measurements were recorded using a stopwatch. The test–retest ICC for this measurement method was 0.93^23)^. Additionally, the inter-rater ICC was 0.81^24)^. The 5mWT was performed by measuring the time taken to walk a 5-m walking path at a fast pace. The walking path had a 3-m reserve path on each side. Shoes were worn while walking. The measurements were acquired twice, and the better of the two results was selected as the representative value. Measurements were recorded using a stopwatch. The test–retest ICC for this measurement method was 0.83^25)^. Additionally, the inter-rater ICC was 0.84^24)^. The JKOM uses a patient-oriented questionnaire comprising 25 items on pain and stiffness, daily living, usual activities, and health status^9)^. A score of 0 corresponds to “no pain, no difficulty,” whereas a score of 4 corresponds to “severe pain, great difficulty.” The total score ranged from 0 to 100, with higher scores indicating more severe symptoms.

### Outcome

The primary outcome measure was the change in HRQOL. The JKOM was used to assess HRQOL in the present study. A change in the JKOM of ≥8 points from the baseline measurement was defined as improvement, whereas a change of <8 points was defined as non-improvement. Previous studies have shown that the change in JKOM in KOA patients who received 8 weeks of exercise therapy ranged from 8 to 14 points^12)^. Although the study period was different in this study, the lower limit of 8 points achieved in 8 weeks was adopted as the cutoff value. This was because an 8-point change in JKOM results in changes in pain and QOL. Patients with a JKOM of <8 points at baseline were excluded. Exercise therapy implemented during conservative treatment mainly comprised a joint range of motion exercises, muscle strengthening, stretching, and cardiac exercises.

### Statistical analysis

The patients were classified into two groups based on the change in JKOM after 5 months (those with a score of ≥8 points were classified into the improved group and those with a score of <8 points were classified into the non-improved group). Next, the descriptive statistics of sex, age, BMI, exercise habits, K-L classification, VAS, bilateral KOA, pharmacological treatment, duration of physical therapy intervention, physical therapy intervention time, physical therapy start date, SER, knee extension strength on the fault side, knee flexion strength on the fault side, knee extension range of motion on the fault side, knee flexion range of motion on the fault side, FTSST, 5mWT, total JKOM score variables, and the test of difference were conducted. Statistical analysis was performed using t-tests and chi-square tests.

These variables were used as the independent variables, while the change in JKOM after 5 months (≥8 and <8 points in the improved and non-improved groups, respectively) was used as the dependent variable, and a decision tree analysis was performed. Non-improvement was defined as the occurrence of an event (coded as 1), and CPR was derived from the nodes of the decision tree. A binary tree was used for the analysis. Branching was terminated if the number of cases was <10 after branching. K-fold cross-validation (k = 10) was used to verify the accuracy of the overall decision tree model. K-fold cross-validation divides the data into K pieces, one of which is the validation data, and the remaining K-1 pieces are the training data, which evaluate the percentage of correct answers; the method is to train K pieces of data one by one for K trials to become the validation data, and then take the average of their accuracy^26)^. The data were split into training and validation data, and 10 trials were performed. The area under the receiver operating characteristic curve (AUROC), classification error rate, sensitivity, specificity, positive and negative predictive values, and positive and negative likelihood ratios were calculated as indices of accuracy. All statistical analyses were performed using JMP Pro Version 17 (SAS Institute Japan, Tokyo, Japan).

### Sample size

The sample size of AUROC and other inspection characteristic values were calculated using the following method. A previous cohort study on patients with KOA reported that 33.8%–47.9% of patients had good HRQOL scores, daily functioning, and the ability to stand and sit^27)^. The ratio was set as 1–2.3, with approximately 70% and 30% in the improved (no events) and non-improved (with events) groups, respectively. Statistical power was set at 0.85 and AUROC at 0.7. Twenty and 46 patients had to be included in the improved and unimproved groups, respectively, to achieve the required sample size.

## Results

**Fig. 1.**
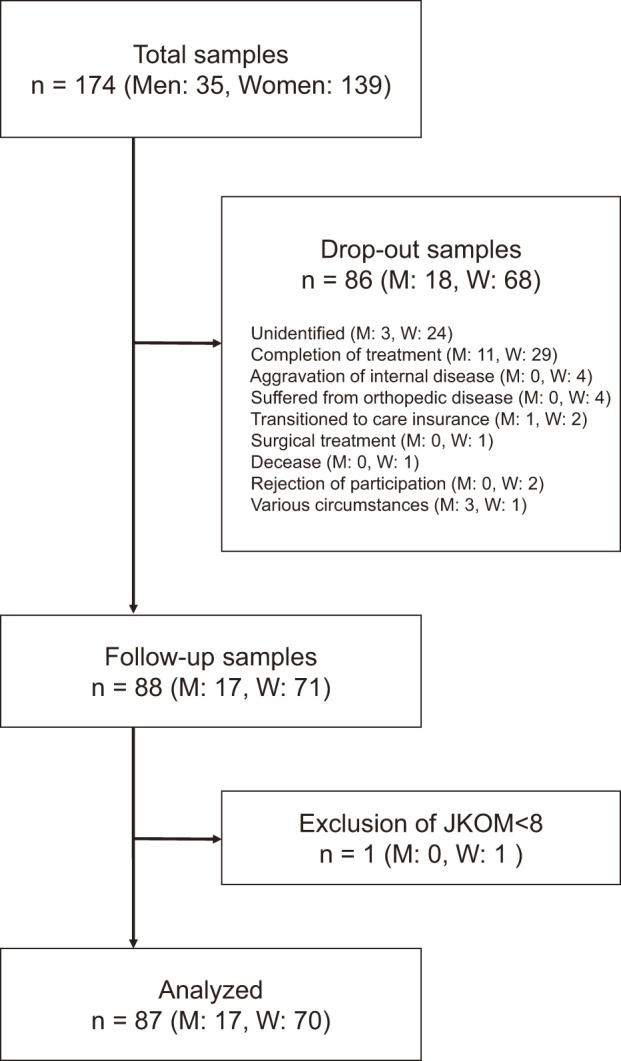
Flowchart depicting the process of selecting the participants to be analyzed. JKOM, Japan Knee Osteoarthritis Measure

A total of 174 participants, comprising 35 men and 139 women, were eligible for inclusion in this study. Among these 174 patients, 88 met the criteria for analysis and were eligible for follow-up for 5 months. Among these 88 patients, one patient was excluded as the JKOM score was <8 at baseline (Fig. 1). Thus, 87 patients were included in the present study. The improved and non-improved groups comprised 28 (32%) and 59 (68%) participants, respectively. The variables that showed significant differences between the improved and non-improved groups were VAS (p = 0.02), duration of physical therapy (p = 0.02), knee extension muscle strength (p = 0.04), 5mWT (p = 0.02), and JKOM (p = 0.002). Table 1 presents the other variables and their details.

**Table 1.** Characteristics of follow-up, drop-out, and exclusion participants

	All (n = 174)	Drop-out and exclusion (n = 87)	Follow-up (n = 87)		p-value
			Improved group (n = 28)	Non-improved group (n = 59)	
Sex	Men: 35, Women: 139	Men: 18, Women: 69	Men: 7, Women: 21	Men: 10, Women: 49	0.94
Age (years)	73.1 (9.0)	72.3 (10.2)	72.3 (10.2)	74.9 (8.6)	0.22
BMI (kg/m^2^)	24.50 (3.82)	25.21 (3.98)	23.48 (4.32)	24.60 (3.86)	0.23
Exercise habits	Yes: 63, No: 111	Yes: 26, No: 61	Yes: 15, No: 13	Yes: 22, No: 37	0.15
K-L classifications	I: 39, II: 60, III: 58, IV: 17	I: 17, II: 27, III: 37, IV: 6	I: 6, II: 12, III: 9, IV: 1	I: 16, II: 21, III: 12, IV: 10	0.68
VAS (mm)	48.00 (25.00)	53.70 (24.16)	50.25 (17.52)	37.61 (25.14)	0.02
Bilateral KOA	Unilateral: 88, Bilateral: 86	Unilateral: 52, Bilateral: 35	Unilateral: 15, Bilateral: 13	Unilateral: 21, Bilateral: 38	0.11
Pharmacological treatment	Yes: 146, No: 28	Yes: 75, No: 12	Yes: 21, No: 7	Yes: 50, No: 9	0.28
Duration of physical therapy	1: 83, 2: 28, 3: 19, 4: 44	1: 61, 2: 8, 3: 10, 4: 8	1: 11, 2: 9, 3: 3, 4: 5	1: 11, 2: 11, 3: 6, 4: 31	0.02
Physical therapy intervention time (min)	58.51 (21.93)	55.81 (19.67)	58.57 (24.90)	63.05 (22.84)	0.41
Physical therapy start date	0: 149, 1: 25	0: 80, 1: 7	0: 26, 1: 2	0: 45, 1: 14	0.06
SER (points)	86.16 (19.67)	84.01 (19.79)	85.57 (19.10)	88.04 (18.33)	0.56
Knee extension muscle strength (Nm/kg)	0.88 (0.41)	0.84 (0.41)	0.98 (0.46)	0.79 (0.35)	0.04
Knee flexion muscle strength (Nm/kg)	0.49 (0.19)	0.48 (0.21)	0.47 (0.18)	0.45 (0.17)	0.60
Knee extension ROM (degree)	-4.77 (4.76)	-4.30 (5.54)	-6.61 (4.72)	-6.52 (4.67)	0.94
Knee flexion ROM (degree)	132.99 (14.35)	132.21 (13.86)	128.57 (15.86)	131.53 (15.95)	0.42
FTSST (sec)	10.24 (4.58)	10.61 (5.05)	9.43 (3.12)	10.13 (4.53)	0.46
5mWT (sec)	4.03 (1.75)	3.97 (2.00)	3.59 (0.89)	4.34 (1.63)	0.02
JKOM (points)	30.14 (16.09)	30.17 (15.27)	38.11 (18.13)	26.73 (14.98)	0.002

Mean (SD). Comparison result between improved and non-improved groups, duration of physical therapy: 1, 1 month or less; 2, 2–5 months; 3, 6–11 months; 4, 12 months or more. Physical therapy intervention time: intervention time per week (minutes). Physical therapy start date: 0, the period from the date of diagnosis to the date of the start of physical therapy is less than 1 month; 1, start of physical therapy is at least 1 month. JKOM <8 to exclude one patient.

BMI, body mass index; K-L, Kellgren-Lawrence; VAS, visual analog scale; KOA, knee osteoarthritis; SER, Self-Efficacy for Rehabilitation Outcome Scale; ROM, range of motion; FTSST, five-times stand and sit test; 5 mWT, 5-m walk test; JKOM, Japan Knee Osteoarthritis Measure

**Fig. 2.**
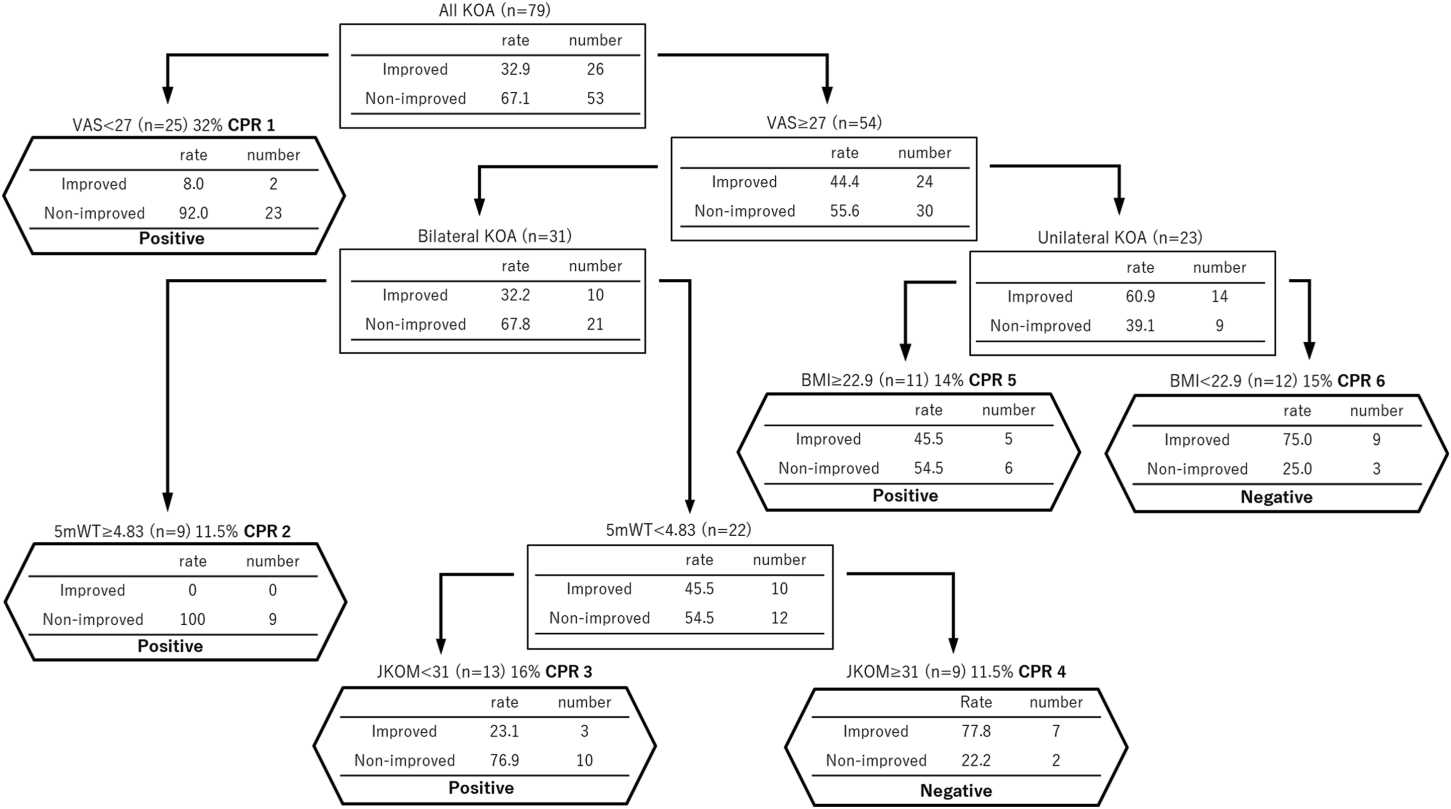
Decision tree model predicting the likelihood of improvement in JKOM after 5 months. ‘Positive’ indicates non-improvement and ‘Negative’ indicates improvement. KOA, knee osteoarthritis; VAS, visual analog scale; CPR, clinical prediction rule; BMI, body mass index; 5mWT, 5-m walk test; JKOM, Japan Knee Osteoarthritis Measure

VAS, bilateral KOA, 5mWT, JKOM, and BMI were the variables derived from the decision tree analysis. Six CPRs were obtained from terminal nodes (Fig. 2). CPR1 comprised a VAS score of <27 mm, which included 32% (25/79) of the participants. CPR2 comprised a VAS score of ≥27 mm, bilateral KOA, and 5mWT of ≥4.83 sec, which included 11.5% (9/79) of participants. CPR3 comprised a VAS score of ≥27 mm, bilateral KOA, 5mWT of <4.83 sec, and JKOM of <31 points, which included 16% (13/79) participants. CPR4 comprised a VAS score of ≥27 mm, bilateral KOA, 5mWT of <4.83 sec, and JKOM of ≥31 points, which included 11.5% (9/79) participants. CPR5 comprised a VAS score of ≥27 mm, unilateral KOA, and BMI of ≥22.9 kg/m^2^, which included 14% (11/79) participants. CPR6 comprised a VAS score of ≥27 mm, unilateral KOA and BMI of <22.9 kg/m^2^, which included 15% (12/79) participants. CPR1, CPR2, CPR3, and CPR5 yielded positive results. Table 2 summarizes the positive and negative results of the CPR (positive indicates non-improvement, and negative indicates improvement). The accuracy validation of the model for the entire decision tree (average of 10 trials) yielded AUROC, classification error rate, sensitivity, specificity, positive predictive ratio, negative predictive ratio, positive likelihood ratio, and negative likelihood ratio of 0.87 (validation data: 0.83), 0.17 (validation data: 0.18), 0.92, 0.64, 0.84, 0.78, 2.6, and 0.1, respectively.

**Table 2.** More about CPRs

Positive CPR
VAS <27 mm (CPR 1)
VAS ≥27 mm, bilateral KOA, 5mWT ≥4.83 sec (CPR 2)
VAS ≥27 mm, bilateral KOA, 5mWT <4.83 sec, JKOM <31 points (CPR 3)
VAS ≥27 mm, unilateral KOA, BMI ≥22.9 kg/m^2^ (CPR 5)
Negative CPR
VAS ≥27 mm, bilateral KOA, 5mWT <4.83 sec, JKOM ≥31 points (CPR 4)
VAS ≥27 mm, unilateral KOA, BMI <22.9 kg/m^2^ (CPR 6)

Average of 10-fold crossing. Positive CPR: non-improvement, negative CPR: improvement.

CPR, clinical prediction rule; VAS, visual analog scale; KOA, knee osteoarthritis; 5mWT, 5-m walk test; JKOM, Japan Knee Osteoarthritis Measure; BMI, body mass index

## Discussion

This study used decision tree analysis to derive a CPR that predicts HRQOL as assessed by the JKOM of patients with KOA after undergoing conservative treatment for 5 months. The variables derived were VAS, bilateral KOA, 5mWT, JKOM, and BMI. The overall accuracy and test characteristic values of the decision tree model were good. In total, there are four positive CPRs and two negative CPRs.

The novelty of the present study is that it derived a CPR that is highly accurate in predicting JKOM in conservatively treated patients with KOA. Limitations of the CPR for KOA in previous studies include a high misclassification rate of 0.39^28)^ and lack of cross-validation^29)^. In this study, a low misclassification rate (training data: 0.17, validation data: 0.18) was confirmed and cross-validation was performed. This confirms the high accuracy of the CPR derived in this study. This derived CPR contributes to the accurate prediction of HRQOL in patients with knee KOA in clinical practice.

The accuracy of the entire classification derived from the decision tree analysis was 0.87 for the AUROC (0.83 for the validation data) and 0.17 for the classification error rate (0.18 for the validation data) for training and validation. AUROC has good accuracy at ≥0.8^30)^. Furthermore, a classification error rate of approximately 0.2 has been commonly reported^31^^,^^32)^. These results were judged to be superior to the reference data. Therefore, similar results are likely to be obtained when this CPR is practiced on other samples of the population. The positive and negative likelihood ratios for CPR in the present study were 2.6 and 0.1, respectively. The change in post-test probability was slight when the positive likelihood ratio ranged from 2 to 5. The change in post-test probability was moderate when the negative likelihood ratio ranged from 0.1 to 0.2^33)^. Thus, a negative CPR (a VAS score of ≥27 mm, bilateral KOA, 5mWT of <4.83 sec, JKOM of ≥31 points, or a VAS score of ≥27 mm, unilateral KOA, BMI of <22.9 kg/m^2^) is considered to rule out the possibility of an event occurring (to derive an improvement group). Increased walking speed in patients with moderate to severe KOA has reportedly led to improved QOL^34)^. CPR with a VAS score of ≥27 mm, bilateral KOA, 5mWT of <4.83 sec, and JKOM of ≥31 points are likely to improve if walking speed is maintained, even in patients with moderate symptoms. Furthermore, a greater number of arthropathies and higher BMI affect activity limitation and arthropathy severity^35^^,^^36)^. In CPR with a VAS score greater than 27 mm, unilateral KOA, and BMI less than 22.9, the symptoms are highly likely to improve because they are mild and there is no metabolic abnormality.

This study used physical function and medical information, such as VAS and 5mWT, to screen patients who should be considered more carefully for exercise therapy. We suggest that screening will allow us to develop a treatment plan for such patients with KOA that includes options other than general exercise therapy. This may contribute to the determination of patients with KOA and the decision to apply treatment options. The clinical utility of this CPR is that when CPR is positive, specific individualized measures should be taken, including measures other than exercise therapy. On the other hand, when CPR is negative, treatment options (local pharmacotherapy, self-management programs, weight control, cognitive-behavioral therapy) following versatile guidelines should be considered as an option^37)^.

This study has four limitations: The first is that 40 of the subjects who had difficulty following up completed treatment earlier than 5 months. It is possible that some patients with KOA had improved by more than 8 points in JKOM within 5 months and that there were more cases in the improved group than in the sample of this study. Therefore, the decision to apply inspection characteristic values such as positive likelihood ratios and negative likelihood ratios should be reserved. Second, in this study, we were able to conduct the training and validation tasks of the CPR but were not able to validate them with test data. Usually, when a model is created, training data, validation data, and test data must be prepared. Therefore, clinicians understand this and should use the findings of this study. To utilize the CPR, it is necessary to collect a separate sample, validate cross-validation, and conduct a budget impact analysis. Third, physical therapy intervention times have not been standardized. The possibility that the time of physical therapy intervention has an interactive effect on the improvement of JKOM must be considered. In the case of clinical application, it is necessary to consider whether the subjects are similar to those in the present study (approximately 60 minutes per week). Fourth, the guarantee of external validity is not sufficient. The age range (73.6 years) and BMI (24.0) of the subjects in this study are similar to those reported in previous studies (age: prevalence increases from 70 years; BMI: 23.0)^38)^. However, the sex ratio in the present study was 20% men and 80% women, while in the previous study, it was 28% men and 72% women^38)^, which is somewhat different. Despite some limitations of this study, it is significant that we derived a CPR that predicts HRQOL at 5 months for patients with KOA undergoing conservative treatment. We believe that this is the first process to utilize this technology in actual clinical practice and will contribute to the development of research areas in the field of KOA.

## Conclusion

This study examined the derivation of a CPR that predicts HRQOL at 5 months for patients with KOA undergoing conservative treatment. The accuracy of the CPR was good for both AUROC and the classification error rate. This CPR was an inspection characteristic that could exclude the possibility of an event occurring due to a negative result. However, since the results of this study represent the first process of utilizing CPR in actual clinical practice, its application should be kept in mind.

## Acknowledgments

We thank the facilities that cooperated in this multi-center joint research.

## Funding

This work was supported by the Grants-in-Aid for Scientific Research of Japan Society for the Promotion of Science (Japan), Grant Number 18K17738.

## Conflicts of Interest

The authors declare no conflicts of interest.
